# Bis(μ-adamantane-1,3-dicarboxyl­ato-κ^4^ *O* ^1^,*O* ^1′^:*O* ^3^,*O* ^3′^)bis­[aqua­(3-carboxy­adam­antane-1-carboxyl­ato-κ*O* ^1^)(1,10-phen­an­throline-κ^2^ *N*,*N*′)erbium(III)] dihydrate

**DOI:** 10.1107/S1600536811023075

**Published:** 2011-06-22

**Authors:** Hong-lin Zhu, Wen-xiang Huang, Hai-sheng Chang

**Affiliations:** a Center of Applied Solid State Chemistry Research, Ningbo University, Ningbo, Zhejiang 315211, People’s Republic of China

## Abstract

The asymmetric unit of the binuclear centrosymmetric title compound, [Er_2_(C_12_H_14_O_4_)_2_(C_12_H_15_O_4_)_2_(C_12_H_8_N_2_)_2_(H_2_O)_2_]·2H_2_O, contains one Er^III^ atom, one coordinated water mol­ecule, one 1,10-phenanthroline (phen) ligand, two differently coordinated adamantane-1,3-dicarboxyl­ate (*H_2_L*) ligands and one lattice water mol­ecule. The Er^III^ ion is eight-coordinated by four O atoms from bridging *L* ^2−^, one O atom from H*L* ^−^, one O atom from the coordinated water and two N atoms from a phen ligand. Extensive O—H⋯O hydrogen-bonding inter­actions result in the formation of chains which are further linked into a layer-like network by π–π stacking inter­actions centroid–centroid distance = 3.611 (3) Å] between adjacent phen ligands belonging to neighbouring chains. The carboxy group of the H*L* ^−^ ligand is equally disordered over two positions.

## Related literature

For 1,3-adamantanedicarb­oxy­lic acid, see: Glidewell & Ferguson (1996 ▶). For lanthanide 1,3-adamantanedicarboxyl­ate complexes, see: Millange *et al.* (2004 ▶); Li *et al.* (2009 ▶).
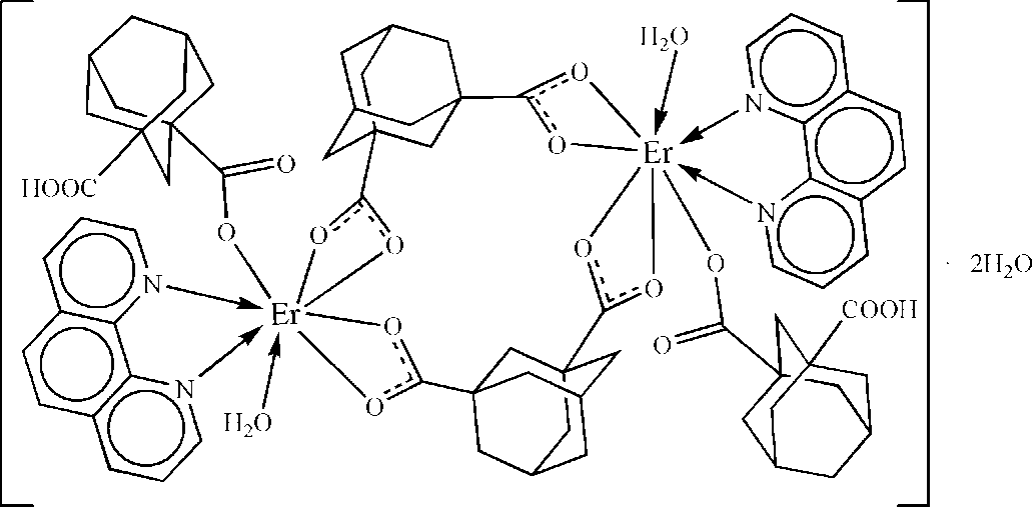


## Experimental

### 

#### Crystal data

[Er_2_(C_12_H_14_O_4_)_2_(C_12_H_15_O_4_)_2_(C_12_H_8_N_2_)_2_(H_2_O)_2_]·2H_2_O
*M* *_r_* = 1657.94
Triclinic, 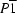

*a* = 8.6164 (17) Å
*b* = 13.579 (3) Å
*c* = 14.560 (3) Å
α = 94.53 (3)°
β = 96.36 (3)°
γ = 92.22 (3)°
*V* = 1685.8 (6) Å^3^
*Z* = 1
Mo *K*α radiation
μ = 2.55 mm^−1^
*T* = 293 K
0.34 × 0.17 × 0.09 mm

#### Data collection

Rigaku R-AXIS RAPID diffractometer
Absorption correction: multi-scan (*ABSCOR*; Higashi, 1995 ▶) *T*_min_ = 0.600, *T*_max_ = 0.795
16743 measured reflections
7645 independent reflections
6761 reflections with *I* > 2σ(*I*)
*R*_int_ = 0.031

#### Refinement

*R*[*F* ^2^ > 2σ(*F* ^2^)] = 0.030
*wR*(*F* ^2^) = 0.067
*S* = 1.07
7645 reflections
448 parameters
H-atom parameters constrained
Δρ_max_ = 0.77 e Å^−3^
Δρ_min_ = −0.45 e Å^−3^

### 

Data collection: *RAPID-AUTO* (Rigaku, 1998 ▶); cell refinement: *RAPID-AUTO*; data reduction: *CrystalStructure* (Rigaku/MSC, 2004 ▶); program(s) used to solve structure: *SHELXS97* (Sheldrick, 2008 ▶); program(s) used to refine structure: *SHELXL97* (Sheldrick, 2008 ▶); molecular graphics: *ORTEPII* (Johnson, 1976 ▶); software used to prepare material for publication: *SHELXL97*.

## Supplementary Material

Crystal structure: contains datablock(s) global, I. DOI: 10.1107/S1600536811023075/jh2296sup1.cif

Structure factors: contains datablock(s) I. DOI: 10.1107/S1600536811023075/jh2296Isup2.hkl

Additional supplementary materials:  crystallographic information; 3D view; checkCIF report

## Figures and Tables

**Table 1:** Hydrogen-bond geometry (Å, °)

*D*—H⋯*A*	*D*—H	H⋯*A*	*D*⋯*A*	*D*—H⋯*A*
O9—H91⋯O10	0.85	1.74	2.558 (4)	161.1
O9—H92⋯O1^i^	0.86	1.92	2.760 (4)	166.7
O10—H101⋯O6	0.85	1.87	2.653 (4)	153.6
O10—H102⋯O4^i^	0.85	1.87	2.692 (4)	161.8

Symmetry code: (i) 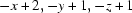.

**Fig. 1.**
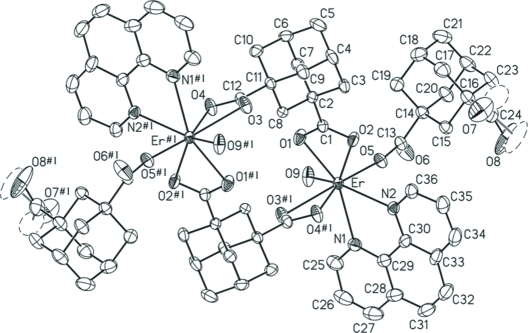
ORTEP view of the title compound. The dispalcement ellipsoids are drawn at 35% probability level. (# = -x + 1, -y + 1, -z + 1)

**Fig. 2.**
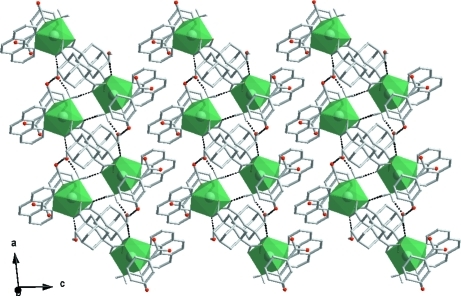
two-dimensional layer-like structure of the title crystal viewed down the b axis. O—H···O hydrogen bonds are shown as dashed line.

## supplementary crystallographic information

### Comment

As known, lanthanide ions have high affinity for hard donor atoms, and ligands with oxygen or hybrid oxygen-nitrogen atoms, especially multicarboxylate ligands are usually employed in construction for lanthanide complexes (Li *et al.*, 2009). Herein, we report the crystal structure of title compound, [Er(phen)(H_2_O)(HL)*L*]_2_.2H_2_O, which consist of 1,3-adamantanedicarboxylic acid (H_2_L), 1,10-phenanthroline and ErCl_3_.nH_2_O. This structure indicates that hydrogen-bond and π–π interaction are responsible for supramolecular assemblies.

The asymmetric unit contains one Er^III^, one coordination water, two type of 1,3-adamantanedicarboxylate ligands and one lattic water. As show in Fig.1, the Er^III^ ion is in a eight coordinated fashion by four oxygen atoms from *L*^2-^, one oxygen from H*L*^1-^, one oxygen from a coordination water and two nitrogen atoms from a 1,10-phenanthroline ligand. Three type kinds of hydrogen-bond are observed in title compound (Table 2). The presence of the extensive hydrogen-bond interaction results in formation of one-dimensional chains, which further grow into two-dimensional layer-like network by π–π stacking interaction between adjacent phen ligands belonging to neighboring chains (Fig. 2).

### Experimental

Pink powder of ErCl_3_.nH_2_O was obtained by slow evaporation of a solution of Er_2_O_3_ (0.150 mmol, 0.0574 g) dissolved in HCl (5 ml) under water boiling condition. A mixture of 1,3-adamantanedicarboxylic acid (0.300 mmol, 0.0595 g) in water (10 ml) was stirred for 30 min, and sealed in a 23 ml Teflon-lined stainless autoclave, which was heated at 170°C for three days and thereafter cooled slowly to room temperature, and pink crystals were separated by filtering and washing.

### Refinement

H atoms bonded to C atoms were palced in geometrically calculated position and were refined using a riding model, with *U*_iso_(H) = 1.2 *U*_eq_(C). H atoms attached to O atoms were found in a difference Fourier synthesis and were refined using a riding model, with the O—H distances fixed as initially found and with *U*_iso_(H) values set at 1.2 *U*eq(O).

### Crystal data

**Table:**

[Er_2_(C_12_H_14_O_4_)_2_(C_12_H_15_O_4_)_2_(C_12_H_8_N_2_)_2_(H_2_O)_2_]·2H_2_O	*Z* = 1
*M**_r_* = 1657.94	*F*(000) = 836
Triclinic, *P*1	*D*_x_ = 1.631 Mg m^−^^3^
Hall symbol: -P 1	Mo *K*α radiation, λ = 0.71073 Å
*a* = 8.6164 (17) Å	Cell parameters from 16743 reflections
*b* = 13.579 (3) Å	θ = 3.0–27.5°
*c* = 14.560 (3) Å	µ = 2.55 mm^−^^1^
α = 94.53 (3)°	*T* = 293 K
β = 96.36 (3)°	Platelet, pink
γ = 92.22 (3)°	0.34 × 0.17 × 0.09 mm
*V* = 1685.8 (6) Å^3^	

### Data collection

**Table:**

Rigaku R-AXIS RAPID diffractometer	7645 independent reflections
Radiation source: fine-focus sealed tube	6761 reflections with *I* > 2σ(*I*)
graphite	*R*_int_ = 0.031
Detector resolution: 0 pixels mm^-1^	θ_max_ = 27.5°, θ_min_ = 3.0°
ω scans	*h* = −9→11
Absorption correction: multi-scan (*ABSCOR*; Higashi, 1995)	*k* = −17→17
*T*_min_ = 0.600, *T*_max_ = 0.795	*l* = −18→18
16743 measured reflections	

### Refinement

**Table:**

Refinement on *F*^2^	Primary atom site location: structure-invariant direct methods
Least-squares matrix: full	Secondary atom site location: difference Fourier map
*R*[*F*^2^ > 2σ(*F*^2^)] = 0.030	Hydrogen site location: inferred from neighbouring sites
*wR*(*F*^2^) = 0.067	H-atom parameters constrained
*S* = 1.07	*w* = 1/[σ^2^(*F*_o_^2^) + (0.0268*P*)^2^ + 1.2992*P*] where *P* = (*F*_o_^2^ + 2*F*_c_^2^)/3
7645 reflections	(Δ/σ)_max_ = 0.001
448 parameters	Δρ_max_ = 0.77 e Å^−^^3^
0 restraints	Δρ_min_ = −0.45 e Å^−^^3^

### Special details

**Table:**

Geometry. All e.s.d.'s (except the e.s.d. in the dihedral angle between two l.s. planes) are estimated using the full covariance matrix. The cell e.s.d.'s are taken into account individually in the estimation of e.s.d.'s in distances, angles and torsion angles; correlations between e.s.d.'s in cell parameters are only used when they are defined by crystal symmetry. An approximate (isotropic) treatment of cell e.s.d.'s is used for estimating e.s.d.'s involving l.s. planes.
Refinement. Refinement of *F*^2^ against ALL reflections. The weighted *R*-factor *wR* and goodness of fit *S* are based on *F*^2^, conventional *R*-factors *R* are based on *F*, with *F* set to zero for negative *F*^2^. The threshold expression of *F*^2^ > σ(*F*^2^) is used only for calculating *R*-factors(gt) *etc*. and is not relevant to the choice of reflections for refinement. *R*-factors based on *F*^2^ are statistically about twice as large as those based on *F*, and *R*- factors based on ALL data will be even larger.

### Fractional atomic coordinates and isotropic or equivalent isotropic displacement parameters (Å^2^)

**Table:**

	*x*	*y*	*z*	*U*_iso_*/*U*_eq_	Occ. (<1)
Er	0.854312 (16)	0.436085 (10)	0.306019 (10)	0.02576 (5)	
O1	0.8026 (3)	0.4032 (2)	0.45688 (17)	0.0447 (7)	
O2	0.6851 (3)	0.30718 (18)	0.34156 (16)	0.0395 (6)	
C1	0.7032 (4)	0.3337 (2)	0.4261 (2)	0.0287 (7)	
C2	0.6055 (4)	0.2855 (2)	0.4926 (2)	0.0283 (6)	
C3	0.4990 (4)	0.1997 (2)	0.4425 (2)	0.0348 (8)	
H3A	0.4298	0.2238	0.3929	0.042*	
H3B	0.5619	0.1498	0.4156	0.042*	
C4	0.4028 (5)	0.1547 (2)	0.5124 (3)	0.0425 (9)	
H4A	0.3359	0.0995	0.4807	0.051*	
C5	0.5110 (5)	0.1166 (3)	0.5897 (3)	0.0540 (11)	
H5A	0.5749	0.0664	0.5642	0.065*	
H5B	0.4499	0.0869	0.6333	0.065*	
C6	0.6162 (5)	0.2030 (3)	0.6399 (3)	0.0426 (9)	
H6A	0.6857	0.1784	0.6900	0.051*	
C7	0.7134 (4)	0.2479 (3)	0.5719 (3)	0.0371 (8)	
H7A	0.7796	0.1986	0.5471	0.044*	
H7B	0.7802	0.3022	0.6034	0.044*	
C8	0.5023 (4)	0.3646 (2)	0.5333 (2)	0.0261 (6)	
H8A	0.5679	0.4198	0.5642	0.031*	
H8B	0.4338	0.3892	0.4837	0.031*	
C9	0.3001 (4)	0.2325 (2)	0.5525 (3)	0.0378 (8)	
H9A	0.2371	0.2034	0.5955	0.045*	
H9B	0.2302	0.2561	0.5029	0.045*	
C10	0.5148 (4)	0.2816 (3)	0.6807 (3)	0.0388 (8)	
H10A	0.4547	0.2534	0.7256	0.047*	
H10B	0.5811	0.3361	0.7123	0.047*	
C11	0.4039 (4)	0.3195 (2)	0.6031 (2)	0.0289 (7)	
C12	0.3065 (4)	0.4003 (2)	0.6422 (2)	0.0314 (7)	
O3	0.1614 (3)	0.39715 (18)	0.6249 (2)	0.0431 (6)	
O4	0.3746 (3)	0.47357 (17)	0.69327 (19)	0.0409 (6)	
O5	1.0009 (3)	0.32069 (17)	0.25012 (19)	0.0390 (6)	
O6	1.2280 (3)	0.2688 (2)	0.2129 (3)	0.0664 (10)	
C13	1.0941 (4)	0.2530 (2)	0.2328 (3)	0.0352 (7)	
C14	1.0322 (4)	0.1460 (2)	0.2345 (3)	0.0334 (7)	
C15	0.8883 (4)	0.1286 (2)	0.1626 (3)	0.0356 (8)	
H15A	0.9174	0.1394	0.1016	0.043*	
H15B	0.8103	0.1754	0.1769	0.043*	
C16	0.8190 (5)	0.0223 (3)	0.1624 (3)	0.0438 (9)	
C17	0.7737 (5)	0.0062 (3)	0.2584 (3)	0.0518 (10)	
H17A	0.6951	0.0520	0.2739	0.062*	
H17B	0.7297	−0.0605	0.2588	0.062*	
C18	0.9169 (5)	0.0224 (3)	0.3297 (3)	0.0540 (11)	
H18A	0.8866	0.0116	0.3912	0.065*	
C19	0.9834 (5)	0.1288 (3)	0.3303 (3)	0.0438 (9)	
H19A	0.9051	0.1748	0.3456	0.053*	
H19B	1.0732	0.1401	0.3769	0.053*	
C20	1.1548 (4)	0.0721 (2)	0.2106 (3)	0.0434 (9)	
H20A	1.2469	0.0826	0.2556	0.052*	
H20B	1.1855	0.0822	0.1498	0.052*	
C21	1.0402 (6)	−0.0498 (3)	0.3064 (4)	0.0630 (13)	
H21A	1.1311	−0.0395	0.3523	0.076*	
H21B	0.9988	−0.1172	0.3074	0.076*	
C22	1.0864 (5)	−0.0340 (3)	0.2113 (3)	0.0536 (11)	
H22A	1.1653	−0.0809	0.1962	0.064*	
C23	0.9438 (5)	−0.0501 (3)	0.1385 (3)	0.0527 (11)	
H23A	0.9015	−0.1175	0.1370	0.063*	
H23B	0.9741	−0.0395	0.0777	0.063*	
C24	0.6791 (5)	0.0091 (3)	0.0904 (3)	0.0540 (11)	
O7A	0.542 (3)	0.0103 (12)	0.1176 (19)	0.105 (5)	0.50
O8A	0.705 (5)	0.037 (5)	0.020 (3)	0.127 (6)	0.50
O7B	0.562 (3)	−0.0412 (12)	0.1035 (19)	0.105 (5)	0.50
O8B	0.687 (5)	0.014 (5)	0.000 (3)	0.127 (6)	0.50
H81	0.6139	0.0253	−0.0385	0.153*	
N1	0.9027 (4)	0.5395 (2)	0.1781 (2)	0.0382 (7)	
C25	1.0004 (5)	0.6182 (3)	0.1918 (3)	0.0511 (10)	
H25A	1.0530	0.6341	0.2508	0.061*	
C26	1.0279 (6)	0.6785 (3)	0.1210 (4)	0.0625 (13)	
H26A	1.0967	0.7337	0.1331	0.075*	
C27	0.9532 (6)	0.6557 (3)	0.0348 (4)	0.0668 (14)	
H27A	0.9709	0.6948	−0.0129	0.080*	
C28	0.8494 (5)	0.5730 (3)	0.0178 (3)	0.0510 (10)	
C29	0.8251 (4)	0.5169 (3)	0.0924 (3)	0.0388 (8)	
C30	0.7153 (4)	0.4327 (3)	0.0783 (2)	0.0378 (8)	
C31	0.7663 (6)	0.5436 (4)	−0.0716 (3)	0.0634 (13)	
H31A	0.7833	0.5796	−0.1217	0.076*	
C32	0.6651 (6)	0.4659 (4)	−0.0844 (3)	0.0640 (13)	
H32A	0.6127	0.4492	−0.1432	0.077*	
C33	0.6346 (5)	0.4071 (3)	−0.0103 (3)	0.0496 (10)	
C34	0.5274 (6)	0.3257 (3)	−0.0201 (3)	0.0592 (12)	
H34A	0.4745	0.3050	−0.0780	0.071*	
C35	0.5011 (5)	0.2774 (3)	0.0544 (3)	0.0525 (10)	
H35A	0.4282	0.2243	0.0488	0.063*	
C36	0.5851 (4)	0.3083 (3)	0.1406 (3)	0.0402 (8)	
H36A	0.5657	0.2747	0.1917	0.048*	
N2	0.6905 (3)	0.3827 (2)	0.1527 (2)	0.0338 (6)	
O9	1.0952 (3)	0.4876 (2)	0.37803 (19)	0.0491 (7)	
H91	1.1783	0.4627	0.3623	0.059*	
H92	1.1200	0.5139	0.4333	0.059*	
O10	1.3385 (3)	0.4392 (3)	0.3024 (2)	0.0754 (12)	
H101	1.3302	0.3787	0.2822	0.090*	
H102	1.4354	0.4554	0.3092	0.090*	

### Atomic displacement parameters (Å^2^)

**Table:**

	*U*^11^	*U*^22^	*U*^33^	*U*^12^	*U*^13^	*U*^23^
Er	0.02433 (7)	0.02698 (7)	0.02613 (8)	0.00104 (5)	0.00666 (5)	−0.00200 (5)
O1	0.0479 (15)	0.0530 (15)	0.0310 (14)	−0.0201 (13)	0.0093 (11)	−0.0047 (11)
O2	0.0471 (14)	0.0451 (13)	0.0251 (12)	−0.0129 (12)	0.0098 (10)	−0.0044 (10)
C1	0.0283 (15)	0.0290 (15)	0.0286 (17)	0.0006 (13)	0.0057 (13)	−0.0018 (12)
C2	0.0317 (16)	0.0274 (14)	0.0266 (16)	0.0026 (13)	0.0076 (13)	0.0011 (12)
C3	0.0405 (18)	0.0298 (15)	0.0344 (19)	−0.0008 (15)	0.0123 (15)	−0.0062 (13)
C4	0.051 (2)	0.0226 (15)	0.054 (2)	−0.0067 (16)	0.0202 (19)	−0.0064 (15)
C5	0.074 (3)	0.0287 (17)	0.067 (3)	0.0111 (19)	0.034 (2)	0.0138 (18)
C6	0.057 (2)	0.0387 (18)	0.037 (2)	0.0179 (17)	0.0118 (18)	0.0140 (15)
C7	0.0410 (19)	0.0342 (17)	0.037 (2)	0.0134 (15)	0.0067 (15)	0.0031 (14)
C8	0.0303 (15)	0.0230 (13)	0.0252 (16)	0.0010 (12)	0.0041 (12)	0.0023 (11)
C9	0.0362 (18)	0.0314 (16)	0.047 (2)	−0.0051 (15)	0.0145 (16)	−0.0034 (15)
C10	0.049 (2)	0.0393 (18)	0.0314 (19)	0.0094 (16)	0.0137 (16)	0.0077 (14)
C11	0.0313 (16)	0.0239 (14)	0.0324 (17)	0.0025 (13)	0.0099 (13)	−0.0009 (12)
C12	0.0309 (16)	0.0300 (15)	0.0346 (18)	0.0024 (14)	0.0125 (14)	−0.0016 (13)
O3	0.0300 (12)	0.0370 (13)	0.0603 (18)	0.0024 (11)	0.0068 (12)	−0.0120 (12)
O4	0.0302 (12)	0.0352 (12)	0.0550 (17)	0.0021 (11)	0.0078 (11)	−0.0159 (11)
O5	0.0355 (13)	0.0301 (11)	0.0510 (16)	0.0065 (10)	0.0061 (11)	−0.0030 (11)
O6	0.0457 (16)	0.0422 (15)	0.115 (3)	−0.0023 (14)	0.0343 (18)	−0.0069 (17)
C13	0.0346 (17)	0.0290 (16)	0.042 (2)	0.0049 (14)	0.0054 (15)	−0.0030 (14)
C14	0.0302 (16)	0.0309 (16)	0.0388 (19)	0.0059 (14)	0.0018 (14)	0.0021 (14)
C15	0.0341 (17)	0.0301 (16)	0.041 (2)	−0.0003 (14)	−0.0005 (15)	0.0023 (14)
C16	0.044 (2)	0.0324 (17)	0.053 (2)	0.0002 (16)	−0.0001 (18)	−0.0009 (16)
C17	0.052 (2)	0.044 (2)	0.061 (3)	−0.0044 (19)	0.014 (2)	0.0074 (19)
C18	0.063 (3)	0.055 (2)	0.047 (2)	0.000 (2)	0.010 (2)	0.0157 (19)
C19	0.048 (2)	0.044 (2)	0.039 (2)	0.0058 (18)	0.0029 (17)	0.0019 (16)
C20	0.0353 (18)	0.0336 (18)	0.060 (3)	0.0086 (16)	0.0033 (18)	−0.0024 (17)
C21	0.069 (3)	0.043 (2)	0.076 (3)	0.003 (2)	−0.009 (3)	0.022 (2)
C22	0.051 (2)	0.0339 (19)	0.076 (3)	0.0162 (18)	0.004 (2)	0.0011 (19)
C23	0.061 (3)	0.0276 (17)	0.068 (3)	0.0018 (18)	0.007 (2)	−0.0054 (17)
C24	0.047 (2)	0.053 (2)	0.058 (3)	−0.013 (2)	0.000 (2)	−0.004 (2)
O7A	0.057 (6)	0.161 (16)	0.092 (8)	−0.030 (11)	−0.012 (5)	0.021 (12)
O8A	0.064 (9)	0.26 (3)	0.039 (13)	−0.042 (10)	−0.024 (8)	−0.014 (11)
O7B	0.057 (6)	0.161 (16)	0.092 (8)	−0.030 (11)	−0.012 (5)	0.021 (12)
O8B	0.064 (9)	0.26 (3)	0.039 (13)	−0.042 (10)	−0.024 (8)	−0.014 (11)
N1	0.0420 (17)	0.0344 (15)	0.0405 (18)	0.0049 (13)	0.0130 (14)	0.0048 (12)
C25	0.051 (2)	0.042 (2)	0.064 (3)	0.0014 (19)	0.020 (2)	0.0091 (19)
C26	0.066 (3)	0.039 (2)	0.089 (4)	0.005 (2)	0.029 (3)	0.018 (2)
C27	0.080 (3)	0.054 (3)	0.080 (4)	0.023 (2)	0.042 (3)	0.031 (2)
C28	0.060 (3)	0.053 (2)	0.048 (2)	0.025 (2)	0.024 (2)	0.0204 (19)
C29	0.0420 (19)	0.0410 (18)	0.038 (2)	0.0188 (16)	0.0156 (16)	0.0078 (15)
C30	0.044 (2)	0.0426 (18)	0.0296 (18)	0.0190 (16)	0.0097 (15)	0.0025 (14)
C31	0.086 (4)	0.072 (3)	0.041 (3)	0.037 (3)	0.023 (2)	0.023 (2)
C32	0.084 (3)	0.079 (3)	0.032 (2)	0.035 (3)	0.007 (2)	0.004 (2)
C33	0.056 (2)	0.066 (3)	0.028 (2)	0.028 (2)	0.0028 (17)	−0.0042 (17)
C34	0.065 (3)	0.068 (3)	0.039 (2)	0.017 (2)	−0.009 (2)	−0.012 (2)
C35	0.046 (2)	0.055 (2)	0.050 (3)	0.004 (2)	−0.0093 (19)	−0.011 (2)
C36	0.0388 (19)	0.0399 (19)	0.039 (2)	0.0026 (16)	−0.0014 (16)	−0.0037 (15)
N2	0.0345 (15)	0.0379 (15)	0.0295 (15)	0.0089 (13)	0.0051 (12)	−0.0006 (12)
O9	0.0301 (13)	0.0638 (17)	0.0483 (17)	0.0018 (13)	0.0023 (12)	−0.0239 (13)
O10	0.0380 (16)	0.094 (2)	0.088 (3)	−0.0218 (17)	0.0258 (16)	−0.048 (2)

### Geometric parameters (Å, °)

**Table:**

Er—O5	2.212 (2)	C16—C17	1.521 (6)
Er—O9	2.277 (3)	C16—C23	1.534 (6)
Er—O2	2.360 (2)	C17—C18	1.519 (6)
Er—O1	2.362 (3)	C17—H17A	0.9700
Er—O4^i^	2.363 (2)	C17—H17B	0.9700
Er—O3^i^	2.419 (2)	C18—C21	1.521 (7)
Er—N1	2.480 (3)	C18—C19	1.532 (5)
Er—N2	2.543 (3)	C18—H18A	0.9800
Er—C1	2.727 (3)	C19—H19A	0.9700
Er—C12^i^	2.763 (3)	C19—H19B	0.9700
O1—C1	1.270 (4)	C20—C22	1.537 (5)
O2—C1	1.246 (4)	C20—H20A	0.9700
C1—C2	1.520 (4)	C20—H20B	0.9700
C2—C7	1.533 (5)	C21—C22	1.512 (7)
C2—C3	1.539 (4)	C21—H21A	0.9700
C2—C8	1.544 (4)	C21—H21B	0.9700
C3—C4	1.530 (5)	C22—C23	1.529 (6)
C3—H3A	0.9700	C22—H22A	0.9800
C3—H3B	0.9700	C23—H23A	0.9700
C4—C5	1.517 (6)	C23—H23B	0.9700
C4—C9	1.526 (5)	C24—O8A	1.16 (6)
C4—H4A	0.9800	C24—O7B	1.23 (3)
C5—C6	1.538 (6)	C24—O7A	1.29 (3)
C5—H5A	0.9700	C24—O8B	1.33 (5)
C5—H5B	0.9700	O8A—H81	1.0865
C6—C7	1.515 (5)	O8B—H81	0.8244
C6—C10	1.531 (5)	N1—C25	1.323 (5)
C6—H6A	0.9800	N1—C29	1.356 (5)
C7—H7A	0.9700	C25—C26	1.401 (6)
C7—H7B	0.9700	C25—H25A	0.9300
C8—C11	1.541 (4)	C26—C27	1.353 (8)
C8—H8A	0.9700	C26—H26A	0.9300
C8—H8B	0.9700	C27—C28	1.396 (7)
C9—C11	1.541 (4)	C27—H27A	0.9300
C9—H9A	0.9700	C28—C29	1.405 (5)
C9—H9B	0.9700	C28—C31	1.434 (7)
C10—C11	1.532 (5)	C29—C30	1.442 (5)
C10—H10A	0.9700	C30—N2	1.356 (5)
C10—H10B	0.9700	C30—C33	1.407 (5)
C11—C12	1.520 (4)	C31—C32	1.330 (7)
C12—O3	1.246 (4)	C31—H31A	0.9300
C12—O4	1.276 (4)	C32—C33	1.433 (6)
C12—Er^i^	2.763 (3)	C32—H32A	0.9300
O3—Er^i^	2.419 (2)	C33—C34	1.401 (7)
O4—Er^i^	2.363 (2)	C34—C35	1.347 (7)
O5—C13	1.272 (4)	C34—H34A	0.9300
O6—C13	1.236 (4)	C35—C36	1.402 (5)
C13—C14	1.530 (4)	C35—H35A	0.9300
C14—C15	1.529 (5)	C36—N2	1.320 (5)
C14—C19	1.533 (5)	C36—H36A	0.9300
C14—C20	1.534 (5)	O9—H91	0.8498
C15—C16	1.541 (5)	O9—H92	0.8558
C15—H15A	0.9700	O10—H101	0.8480
C15—H15B	0.9700	O10—H102	0.8481
C16—C24	1.503 (6)		
			
O5—Er—O9	78.78 (9)	O6—C13—O5	124.0 (3)
O5—Er—O2	87.54 (9)	O6—C13—C14	119.1 (3)
O9—Er—O2	128.70 (11)	O5—C13—C14	116.9 (3)
O5—Er—O1	109.70 (10)	C15—C14—C13	108.1 (3)
O9—Er—O1	83.85 (10)	C15—C14—C19	108.6 (3)
O2—Er—O1	54.79 (8)	C13—C14—C19	110.3 (3)
O5—Er—O4^i^	154.07 (9)	C15—C14—C20	108.9 (3)
O9—Er—O4^i^	125.76 (9)	C13—C14—C20	111.6 (3)
O2—Er—O4^i^	82.43 (9)	C19—C14—C20	109.2 (3)
O1—Er—O4^i^	83.74 (10)	C14—C15—C16	110.4 (3)
O5—Er—O3^i^	148.03 (8)	C14—C15—H15A	109.6
O9—Er—O3^i^	71.80 (9)	C16—C15—H15A	109.6
O2—Er—O3^i^	120.65 (9)	C14—C15—H15B	109.6
O1—Er—O3^i^	79.70 (10)	C16—C15—H15B	109.6
O4^i^—Er—O3^i^	54.06 (8)	H15A—C15—H15B	108.1
O5—Er—N1	90.47 (10)	C24—C16—C17	111.2 (3)
O9—Er—N1	88.61 (11)	C24—C16—C23	110.3 (3)
O2—Er—N1	141.19 (10)	C17—C16—C23	109.4 (4)
O1—Er—N1	156.51 (10)	C24—C16—C15	108.2 (3)
O4^i^—Er—N1	82.73 (10)	C17—C16—C15	109.1 (3)
O3^i^—Er—N1	76.82 (10)	C23—C16—C15	108.6 (3)
O5—Er—N2	79.96 (10)	C18—C17—C16	109.8 (3)
O9—Er—N2	146.34 (10)	C18—C17—H17A	109.7
O2—Er—N2	75.75 (9)	C16—C17—H17A	109.7
O1—Er—N2	128.10 (9)	C18—C17—H17B	109.7
O4^i^—Er—N2	74.42 (9)	C16—C17—H17B	109.7
O3^i^—Er—N2	119.14 (10)	H17A—C17—H17B	108.2
N1—Er—N2	65.76 (10)	C17—C18—C21	110.0 (4)
O5—Er—C1	100.85 (10)	C17—C18—C19	109.4 (4)
O9—Er—C1	108.07 (11)	C21—C18—C19	109.8 (4)
O2—Er—C1	27.15 (9)	C17—C18—H18A	109.2
O1—Er—C1	27.72 (9)	C21—C18—H18A	109.2
O4^i^—Er—C1	80.62 (10)	C19—C18—H18A	109.2
O3^i^—Er—C1	99.93 (9)	C14—C19—C18	109.5 (3)
N1—Er—C1	161.29 (10)	C14—C19—H19A	109.8
N2—Er—C1	101.33 (10)	C18—C19—H19A	109.8
O5—Er—C12^i^	170.85 (9)	C14—C19—H19B	109.8
O9—Er—C12^i^	98.32 (10)	C18—C19—H19B	109.8
O2—Er—C12^i^	100.90 (9)	H19A—C19—H19B	108.2
O1—Er—C12^i^	78.40 (10)	C14—C20—C22	109.7 (3)
O4^i^—Er—C12^i^	27.44 (9)	C14—C20—H20A	109.7
O3^i^—Er—C12^i^	26.80 (9)	C22—C20—H20A	109.7
N1—Er—C12^i^	80.75 (10)	C14—C20—H20B	109.7
N2—Er—C12^i^	98.56 (10)	C22—C20—H20B	109.7
C1—Er—C12^i^	88.30 (10)	H20A—C20—H20B	108.2
C1—O1—Er	92.4 (2)	C22—C21—C18	109.4 (4)
C1—O2—Er	93.07 (19)	C22—C21—H21A	109.8
O2—C1—O1	119.4 (3)	C18—C21—H21A	109.8
O2—C1—C2	121.0 (3)	C22—C21—H21B	109.8
O1—C1—C2	119.6 (3)	C18—C21—H21B	109.8
O2—C1—Er	59.78 (17)	H21A—C21—H21B	108.2
O1—C1—Er	59.92 (17)	C21—C22—C23	110.2 (4)
C2—C1—Er	173.5 (2)	C21—C22—C20	109.6 (4)
C1—C2—C7	109.6 (3)	C23—C22—C20	109.0 (3)
C1—C2—C3	111.3 (3)	C21—C22—H22A	109.4
C7—C2—C3	110.0 (3)	C23—C22—H22A	109.4
C1—C2—C8	108.1 (2)	C20—C22—H22A	109.4
C7—C2—C8	109.0 (3)	C22—C23—C16	109.6 (3)
C3—C2—C8	108.8 (3)	C22—C23—H23A	109.8
C4—C3—C2	109.0 (3)	C16—C23—H23A	109.8
C4—C3—H3A	109.9	C22—C23—H23B	109.8
C2—C3—H3A	109.9	C16—C23—H23B	109.8
C4—C3—H3B	109.9	H23A—C23—H23B	108.2
C2—C3—H3B	109.9	O8A—C24—O7B	126 (2)
H3A—C3—H3B	108.3	O8A—C24—O7A	122 (3)
C5—C4—C9	110.0 (3)	O7B—C24—O7A	34.3 (12)
C5—C4—C3	109.9 (3)	O8A—C24—O8B	18 (5)
C9—C4—C3	109.8 (3)	O7B—C24—O8B	111 (2)
C5—C4—H4A	109.1	O7A—C24—O8B	117 (2)
C9—C4—H4A	109.1	O8A—C24—C16	112.4 (18)
C3—C4—H4A	109.1	O7B—C24—C16	120.9 (13)
C4—C5—C6	109.4 (3)	O7A—C24—C16	118.4 (12)
C4—C5—H5A	109.8	O8B—C24—C16	123.7 (19)
C6—C5—H5A	109.8	C24—O8A—H81	118.2
C4—C5—H5B	109.8	C24—O8B—H81	126.2
C6—C5—H5B	109.8	C25—N1—C29	118.7 (3)
H5A—C5—H5B	108.3	C25—N1—Er	121.6 (3)
C7—C6—C10	109.6 (3)	C29—N1—Er	119.8 (2)
C7—C6—C5	109.6 (3)	N1—C25—C26	122.6 (5)
C10—C6—C5	109.6 (3)	N1—C25—H25A	118.7
C7—C6—H6A	109.3	C26—C25—H25A	118.7
C10—C6—H6A	109.3	C27—C26—C25	119.2 (4)
C5—C6—H6A	109.3	C27—C26—H26A	120.4
C6—C7—C2	109.7 (3)	C25—C26—H26A	120.4
C6—C7—H7A	109.7	C26—C27—C28	119.8 (4)
C2—C7—H7A	109.7	C26—C27—H27A	120.1
C6—C7—H7B	109.7	C28—C27—H27A	120.1
C2—C7—H7B	109.7	C27—C28—C29	118.0 (4)
H7A—C7—H7B	108.2	C27—C28—C31	123.1 (4)
C11—C8—C2	109.9 (2)	C29—C28—C31	118.9 (4)
C11—C8—H8A	109.7	N1—C29—C28	121.8 (4)
C2—C8—H8A	109.7	N1—C29—C30	118.6 (3)
C11—C8—H8B	109.7	C28—C29—C30	119.7 (4)
C2—C8—H8B	109.7	N2—C30—C33	122.3 (4)
H8A—C8—H8B	108.2	N2—C30—C29	118.0 (3)
C4—C9—C11	109.6 (3)	C33—C30—C29	119.7 (4)
C4—C9—H9A	109.8	C32—C31—C28	121.3 (4)
C11—C9—H9A	109.8	C32—C31—H31A	119.4
C4—C9—H9B	109.8	C28—C31—H31A	119.4
C11—C9—H9B	109.8	C31—C32—C33	122.0 (4)
H9A—C9—H9B	108.2	C31—C32—H32A	119.0
C11—C10—C6	109.8 (3)	C33—C32—H32A	119.0
C11—C10—H10A	109.7	C34—C33—C30	117.5 (4)
C6—C10—H10A	109.7	C34—C33—C32	124.0 (4)
C11—C10—H10B	109.7	C30—C33—C32	118.5 (4)
C6—C10—H10B	109.7	C35—C34—C33	119.9 (4)
H10A—C10—H10B	108.2	C35—C34—H34A	120.0
C12—C11—C10	110.4 (3)	C33—C34—H34A	120.0
C12—C11—C9	111.6 (3)	C34—C35—C36	119.1 (4)
C10—C11—C9	109.5 (3)	C34—C35—H35A	120.5
C12—C11—C8	107.9 (2)	C36—C35—H35A	120.5
C10—C11—C8	108.6 (3)	N2—C36—C35	123.2 (4)
C9—C11—C8	108.8 (3)	N2—C36—H36A	118.4
O3—C12—O4	119.0 (3)	C35—C36—H36A	118.4
O3—C12—C11	121.5 (3)	C36—N2—C30	118.0 (3)
O4—C12—C11	119.4 (3)	C36—N2—Er	124.2 (2)
O3—C12—Er^i^	61.06 (17)	C30—N2—Er	117.8 (2)
O4—C12—Er^i^	58.57 (16)	Er—O9—H91	122.1
C11—C12—Er^i^	171.5 (2)	Er—O9—H92	128.7
C12—O3—Er^i^	92.14 (19)	H91—O9—H92	105.2
C12—O4—Er^i^	94.0 (2)	H101—O10—H102	106.4
C13—O5—Er	169.9 (3)		

Symmetry codes: (i) −*x*+1, −*y*+1, −*z*+1.

### Hydrogen-bond geometry (Å, °)

**Table:**

*D*—H···*A*	*D*—H	H···*A*	*D*···*A*	*D*—H···*A*
O9—H91···O10	0.85	1.74	2.558 (4)	161.1
O9—H92···O1^ii^	0.86	1.92	2.760 (4)	166.7
O10—H101···O6	0.85	1.87	2.653 (4)	153.6
O10—H102···O4^ii^	0.85	1.87	2.692 (4)	161.8

Symmetry codes: (ii) −*x*+2, −*y*+1, −*z*+1.
